# Towards organizational development for sustainable high-quality medical teaching

**DOI:** 10.1007/s40037-013-0043-6

**Published:** 2013-02-01

**Authors:** Rik Engbers, Léon I. A. de Caluwé, Paul M. J. Stuyt, Cornelia R. M. G. Fluit, Sanneke Bolhuis

**Affiliations:** 1 Department for Evaluation, Quality and Development of Medical Education, Radboud University Nijmegen Medical Centre, 306 IWOO, P.O. Box 9101, 6500 HB Nijmegen, the Netherlands; 2 Department of Management & Organization, Faculty of Economics and Business Administration, VU University Amsterdam, De Boelelaan 1105, 1081 HV Amsterdam, the Netherlands

**Keywords:** Faculty development, Organizational development, Medical teaching

## Abstract

Literature shows that faculty development programmes are not organizationally embedded in academic hospitals. This leaves medical teaching a low and informal status. The purpose of this article is to explore how organizational literature can strengthen our understanding of embedding faculty development in organizational development, and to provide a useful example of organizational development with regards to medical teaching and faculty development. Constructing a framework for organizational development from the literature, based on expert brainstorming. This framework is applied to a case study. A framework for organizational development is described. Applied in a context of medical teaching, these organizational insights show the process (and progress) of embedding faculty development in organizational development. Organizational development is a necessary condition for assuring sustainable faculty development for high-quality medical teaching. Organizational policies can only work in an organization that is developing. Recommendations for further development and future research are discussed.

## Introduction

Improving the quality of teaching in academic higher education can be difficult, because of the competing demands to do research. The demands of patient care and service delivery in academic hospitals make developing a career in academic medical teaching even more difficult [1]. At academic hospitals, medical teachers identify themselves first and foremost as physicians. The profession of doctor has institutionalized over the past two centuries and the doctor’s position in the hospital is firmly established. Most academic hospitals have established organizational support for physicians’ professional role as researcher too, including policies and incentives. But for the professional role of medical teacher, these carrots and sticks are usually lacking, leaving medical teaching a low and informal status. Academic hospitals foster high-quality medical teaching, but physicians’ professional role as medical teacher is not formalized, and it has no explicitly defined academic status.

For over three decades, faculty development programmes have been designed to promote quality and excellence in medical teaching through the development of individual staff, but usually without supporting organizational development [2, 3]. O’Sullivan and Irby [4] state that research on faculty development ‘has produced relatively little generalizable knowledge that can guide faculty development programmes.’ Wilkerson and Irby [5] were the first to state that, besides measures aimed at individual teaching skills, institutional policies are required to promote academic excellence. Thirteen years later, the next steps in faculty development for medical teachers still include ‘identifying funding for and recognition of faculty development’ [6]. Although the focus of faculty development in medical education and the ways to evaluate its outcomes have broadened [7–10], it is still largely about individual teachers, methods and content. Common challenges and future directions concerning faculty development are identified, but not much is known about how to organizationally embed a faculty development programme. Future directions include developing alternative models of faculty development, situating faculty development in a theoretical context, and moving beyond teaching and instructional improvement [11]. Bligh and Brice [1] make some recommendations for medical teaching to develop professionally within an organization (e.g., career incentives, rewarding excellence, and setting professional standards). However, as far as we know, this integrated view on faculty development and organizational development has not yet been addressed extensively in the literature. Therefore, we explored the organizational literature, and brainstormed with an expert to see how it can strengthen our understanding of embedding faculty development in organizational development in academic hospitals. Then we describe a case and analyze it in the light of these organizational insights in order to make recommendations for further development.

## Insights from organizational literature

To frame organizational development as a concept, we looked for characteristics of professional organizations that are related to the integration and stimulation of diverse professional roles.

First, we scanned the literature on faculty development over the last 30 years. Second, we searched for “Faculty development” and “Organizational development” in PubMed, but none of the articles found offered an extensive integrated view on faculty development and organizational development. Because perspectives on organizational development concerning medical teaching are scarce in medical education literature, we decided to look for expertise in the organizational world, and included an author (LC) who is a renowned expert in organizational change management. To frame organizational development as a concept, we looked for characteristics of professional organizations that are related to the integration and stimulation of diverse professional roles. We brainstormed on different points of view on organizations, and came up with four useful perspectives that can be used as a framework to look at academic hospitals and analyze their organizational development concerning medical education. Then we used the expertise and experience of all the authors to determine the leading concepts concerning the four points of view, and focused on the ‘founders’ of those concepts for literature.

To look at the process of organizational change, the authors decided to use Lewin’s [12] three-phase theory for organizational change. This model, still often used today in organization sciences, involves unfreezing, moving, and refreezing. Unfreezing is about helping stakeholders understand that change is required. Moving is about the process of change, the actual organizational development. Refreezing is about making the change permanent, the institutionalization of organizational changes [13]. It shows that institutionalization happens through the process of organizational development. If we apply this model to medical education, it means that for the professional role of medical teacher to develop from a low and informal status to a formal status and to eventually become institutionalized, organizational development initiatives are needed.

### The structure of professional organizations

For a professional organization to develop, it is helpful to determine the structure of the organization. A concept of a structure that can be useful here is what the organization sciences call the professional bureaucracy [14]. Professional bureaucracies rely on standardized skills. Universities and hospitals often show this structure: they work like bureaucracies, but they need highly trained staff to deliver their services. Thus, employees have a large autonomy. The organization has a relatively flat hierarchy, where professionals, accredited through external institutions, do the central work. Another name for this kind of organization is a professional service firm [15, 16]: their core business consists of independent, highly skilled and educated people performing knowledge work and providing non-routine services in close interaction with their clients or client organizations.

This structure fits best into a complex but fairly stable environment. This type of organization is good at executing (complex) state-of-the-art tasks but not as adept when it comes down to changing them [17]. When they have great expertise in a task and a new approach comes up, they do not know how to integrate the change and the normal professional strategy is one of exclusion and non-change. This may be especially applicable to the medical teaching role, which has changed and has become much more demanding than before. Additional organizational measures seem necessary to stimulate the integration and innovation of this role.

### Multiple ladder system for core competencies development

An interesting idea for the development of a professional organization is the multiple ladder system [18]. It assumes that individuals can grow and develop in a variety of competencies and especially in those that are essential to the organization: its core competencies, for instance research competencies, teaching competencies, commercial competencies, and management competencies. Each competency has a ladder with steps for development. The professional at the top performs the most complicated tasks and can act as mentor or coach for those lower on the ladder. The ones lower on the ladder perform less complex activities and can learn to become better. This system allows an organization to foster its core competencies and to develop people in these competencies. It requires formulating levels of mastery of the competencies and defining a variety of career lines.

### The HR approach to organizational development

Another way to develop a professional organization is through interventions on the Human Resources aspect of a (professional) organization [19]. Two well-known ways to do this are:

- Performance Management, which is concerned with managing individual, group and organization performance. It involves goal setting, performance appraisal and reward systems that align work behaviour with business strategy, employee development and workplace technology. Goal setting describes the interaction between managers and employees in jointly defining work behaviours and outcomes. Performance appraisal is a systematic process to assess work-related achievements, strengths and weaknesses. It can also facilitate career counselling, provide information about strengths and diversity of human resources in the company and link employee performance with rewards. Reward systems are concerned with eliciting and reinforcing desired behaviours and work outcomes through compensation and other forms of recognition. A large diversity of innovative and effective reward systems is used in organizations today.
- Developing Talent contains three approaches. Coaching and mentoring, which is aimed at improving skills, knowledge and capabilities of one or more people in the organization. Career planning and development, which addresses different professional needs and concerns as members of the organization progress through their working lives. Third: management and leadership development processes that attempt to transfer knowledge and skills to many individuals. They can be: in-house training programmes, external educational opportunities, action learning projects etc.

### Professional learning in communities of practice

Medical professionals (learn to) interpret and perform their role in medical teaching within the social context of their job. The groups with which they identify themselves can be regarded as communities of practice. The concept of communities of practice (CoP) focuses not on teaching, but on learning as an aspect of functioning in a social context. Wenger [20, 21] sees learning as a process deeply embedded in social learning systems. CoPs are informal social groups and as such often blur the boundaries of organizational contexts or departments. Each CoP defines—usually implicitly—what constitutes competencies. These competencies are defined by three elements:

1. Sense of joint enterprise: members understand and share what their CoP is about and how they can contribute.
2. Relationships of mutuality: communities are built and sustained through mutual interaction between their members.
3. Shared repertoire: CoPs produce a common history and a common repertoire of stories, languages, artifacts, routines, rituals and processes.

The establishment of a community implies the establishment of boundaries (who belongs or does not belong to the community). However, for CoPs to innovate, boundaries need to be spanned and transgressed to facilitate the flow of (new) information. Wenger, McDermott and Snyder [21] argue that CoPs can become hostage to their own history: routines, habits, shared repertoire and fixed patterns make development difficult. There are three ways of managing these boundaries: through people (as brokers between CoPs) [22]; through artifacts (tools, documents, models) and through interaction (challenging your ideas with another community).

### A framework for professional organizational development

**Fig. 1:**
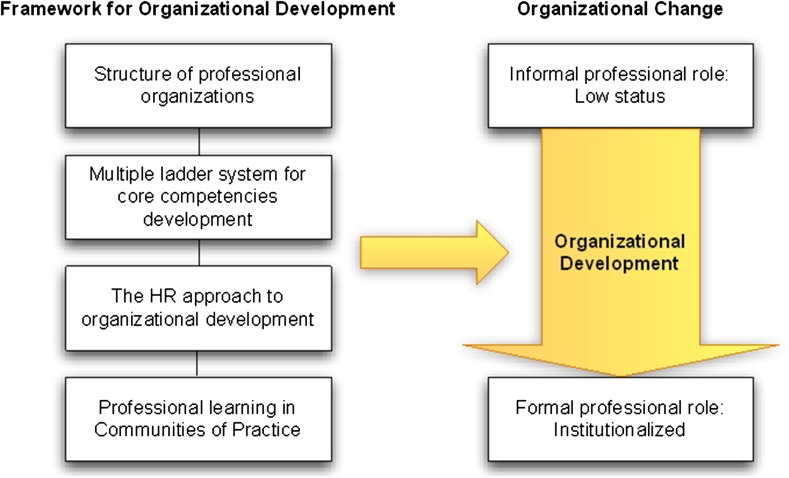
A framework for professional organizational development towards sustainable high-quality medical teaching

A framework to look at organizational change and the professional organizational development of academic hospitals is constructed from organization sciences literature and summarized in Fig. 1. We think that faculty development initiatives (that improve the quality of medical teaching) will only sustain when they are placed within the context of organizational development. The framework in Fig. 1 will be used to look at the status of medical teaching in a case study, laying out perspectives for improvement and sustainability (institutionalization).

## Case description

In Box 1 we describe the case of the Radboud University Nijmegen Medical Centre (RUNMC), located in the east of the Netherlands. University Medical Centres are academic hospitals with a medical research task as well as a large educational task. In the Netherlands, students enter one of eight University Medical Centres after high school for a bachelor and master programme (6 years total). Each year, 300–400 students enter the RUNMC. In Box 1, the organizational development initiatives (a–d) and the faculty development initiatives (e–g) at the RUNMC are described. This case is the object of our analysis. In the analysis we use the framework we took from organization literature. We look at how the organizational development and faculty development initiatives strengthen each other in promoting a sound position of medical education. The RUNMC is not only ‘diagnosed’ using the framework, the framework is also used to provide ‘therapy’ in the form of suggestions for further development and improvement.

**Box 1:** Organizational and Faculty initiatives at the RUNMC

**Organizational development**
*(a) A system of teacher qualifications*
The RUNMC has developed and implemented a unique framework of teaching competencies, focused on the preclinical and clinical (setting of) medical education. All teachers are required, helped by a coach (a trained peer), to obtain a teacher qualification by completing an organized track, including the construction of an educational portfolio. There are four qualification levels (start, basic, extended, full), fitting different teaching roles in three types of education (theoretical education, clinical education, and research internships). Qualification plays a role in the Educational Service Level Agreements and in the (Junior) Principle Lecturer system as discussed below.
*(b) Educational service level agreements*
Educational Service Level Agreements are made between the Dean of the Faculty and the departments, and addressed in quarterly meetings. A future-oriented staffing table for education is established, giving an overview of the needed teachers (and their teacher qualifications) for the education within the department, now and for the upcoming years.
*(c) (Junior) Principal lecturer predicates*
Teachers who have a leading role in education can be awarded a (Junior) Principal Lecturer predicate, which means a teacher can become a professor based on an outshining role in medical education. Also, the (J)PL predicates bring a financial bonus for the department of the teacher.
*(d) Subsidized innovation and research projects in medical education*
As of 2011, departments can apply yearly for funding that is awarded to encourage innovation and research projects in the field of medical education.
**Faculty development**
*(e) Teach the teacher courses*
Teachers at different levels need various types of training [23]. Teach the teacher courses are offered in all three types of education described within the system of Teacher qualifications.
*(f) Guided reflective group sessions and coaching for teachers*
*Coaching*
Coaching to professionalize teaching is offered in two ways:
(1) During the track to obtain a teacher qualification
A teacher who wants to obtain a (higher) teacher qualification follows a track with a coach.
(2) Professional coaching on a specific coaching question or learning objective
Teachers, whether or not they are on a track to a higher teacher qualification, can also get professional coaching in education, focused on a specific learning objective.
*Guided reflective group sessions*
During guided reflective group sessions, teachers exchange questions that relate to their teaching practice. For some groups of teachers in parts of the curriculum (e.g., during clerkships) attending these sessions at set times is obligatory.
*(g) Peer review*
Many forms of peer review are present in a track for obtaining a teacher qualification. Teachers work on their learning objectives of their personal development plan, and discuss their approach and progress with the trajectory coach (a peer). In a number of courses, peer observation of teaching is implemented.

## Case analysis

### The structure of professional organizations

If one looks at the RUNMC as a professional organization, the organizational structure fits the description of a professional service firm or professional bureaucracy. The faculty development initiatives offered at the RUNMC are strengthened by four organizational development initiatives (a–d in Box 1). These organizational development initiatives may be merely the starting point for organizational change. In the current situation, the developed expertise is mainly medical, and research skills are more established than skills in medical teaching. Although medical teaching is as old as the doctor’s profession, it is a relatively new field of additional professional development, compared with the roles of doctor and researcher, which have been institutionalized for a long time. To develop an organization like the RUNMC to institutionalize medical teaching as a full professional role, similar processes are necessary. To develop into a multiple professional services firm, developing all core professional roles of patient care, medical research and medical education, leadership to achieve organizational development is necessary.

### Multiple ladder system for core competencies development

We have observed that the RUNMC has the structure of a professional service firm or professional bureaucracy, and has only just begun to develop as a professional organization in the field of medical education. When looking at how the multiple ladder system is applied at the RUNMC with regards to medical teaching, we see that the RUNMC needs employees with three core competencies: patient care, medical teaching and research [24]. The multiple ladder system usually applies to research: PhD students are coached by supervisors and co-supervisors, Associate Professors and Professors. The multiple ladder system also seems to apply to medical education in the RUNMC, as the systems of Teacher Qualifications and of (Junior) Principal Lecturer Predicates both consist of a detailed complete framework of competencies. For all Teacher Qualification levels in each type of education, a ladder with steps for development is created. The teachers lower on the ladder do simpler teaching activities, and can learn to improve. Also, all teachers in the process of obtaining a higher teacher qualification are helped by a coach, a trained peer, who acts as a mentor for the teacher lower on the ladder. The multiple ladder system can also be observed in the clerkships postgraduate medical education, as specialists in their role as supervisor coach residents and residents in turn often coach medical students.

Today, the multiple ladder system can be recognized in medical teaching at the RUNMC, offering a structure for HR development. This system needs to be persisted and expanded for the status of medical teaching to develop further towards future institutionalization.

### The HR approach to organizational development

If the RUNMC is to develop as a professional organization, interventions on the Human Resource aspect need to be possible. We use this perspective to determine how two well-known ways to intervene at this level, performance management and talent development, are fostered.

#### Performance management

- *Goal setting:* In the Educational Service Level Agreements between the Dean of the Faculty and the departments, goals are set. It is agreed upon what education the department takes care of. The heads of department and the employees, the teachers, annually define their work outcomes together. The teachers’ Teaching Qualifications are discussed, and the ambition to work towards a (Junior) Principal Lecturer Predicate or to apply for a subsidized Innovation and Research Project in Medical Education.
- *Performance appraisal:* Departments’ educational performance is appraised annually, including a quarterly check-up with the Board of Directors. Within the departments, the teachers’ strengths and weaknesses are systematically assessed in annual meetings with their head of department. During the track towards a Teaching Qualification, instruments are provided to reflect on one’s own strengths and weaknesses. The results are then discussed with a coach, and a plan to address the weaknesses is made. Also, peer review and guided reflective group sessions provide useful performance information, and teach the teacher courses as well as professional coaching/counselling are available for all teachers.
- *Reward systems:* The reward systems used at the RUNMC include the system of Teacher Qualifications and (Junior) Principal Lecturer Predicates, offering status and financial grants. Departments that meet the Educational Service Level Agreements do not have to face any educational budget cuts, and the Teacher Qualifications are conditional for a career in medical teaching.

#### Developing talent

- *Coaching and mentoring:* Teachers are coached during the track towards Teaching Qualifications. Peer review, guided reflective group sessions, teach the teacher courses, and professional coaching are available for all teachers at the RUNMC, all aimed at improving the skills, knowledge and capabilities of all teachers.
- *Career planning and development:* Different professional needs and concerns are met at the RUNMC, offering career incentives (Teacher Qualifications and (Junior) Principal Lecturer Predicates) and rewarding excellence. Teach the teacher courses and professional coaching are available to all teachers, and more importantly, annually their career and development is discussed, and tailored agreements are made for patient care, medical teaching and research.
- *Management and leadership development processes:* With the Teacher Qualifications and (Junior) Principal Lecturer Predicates, new leaders in medical teaching emerge. The associated financial bonus and scientific recognition have a trickledown effect, boost research in medical education (which is also separately rewarded in a grant round every year), and offer the opportunity for leadership to develop within the field of medical education. The multiple ladder system that gives every teacher a fitting place in education and offers growth incentives makes that all teachers are easily included.

There are good possibilities to intervene on the Human Resource aspect at the RUNMC. With this structure in place, it is important for leaders/managers of departments to acknowledge and support the position of medical education. Therefore, medical education should, for instance, be included in leadership development initiatives at the HR level.

### Professional learning in communities of practice

There are many different CoPs (some visible, some invisible) within the RUNMC. As doctors identify themselves first and foremost as doctors, most likely they primarily feel members of CoPs that are related to their tasks in patient care. To support identity development as a medical teacher, it seems important to encourage CoPs around medical education. Managing the boundaries between the CoPs is the key to facilitating new learning. Educationalists within the RUNMC act as the brokers between the CoPs, facilitating medical teachers, and helping them to get and stay connected with each other. Medical teachers from different medical CoPs come together during the faculty development initiatives, and are stimulated to form new medical education CoPs. Also the organizational development initiatives facilitate the flow of new information, bringing together and creating new CoPs of (Junior) Principal Lecturers and researchers in medical education.

Artifacts are also present to manage boundaries: the complete framework of teaching competencies laid out for all qualification levels in each type of education is a detailed professional standard, as are the detailed competency profiles for the system of (Junior) Principal Lecturer Predicates. All policies, methods and materials are distributed through the website, open to members of all CoPs.

Though interaction between CoPs is facilitated at the RUNMC, the value of participation could be made more visible and facilitated more structurally. Challenging ideas with another community is a powerful way for professionals to learn. Gathering educational leaders (e.g., Principal Lecturers) in new CoPs is recommended, to discuss medical education research projects and to create and share innovations in the field of medical education [25]. Also, organizing more (structural) meetings for exchanging how to do research on medical teaching is recommended.

## Conclusion and Discussion

The professional development of academic hospitals towards sustainable high-quality medical teaching is a very complex challenge, and should be considered, next to being a strategic cultural change, as an organizational transformation [19]. A framework to look at the professional organizational development of academic hospitals was provided from organization sciences literature and summarized in Fig. 1. The case study has shown that, according to the framework for organizational development, medical teaching at the RUNMC is moving away from its low and informal status, as organizational initiatives are supporting a development towards a position of medical teaching similar to medical research. For the professional role of the medical teacher to develop and to ultimately become institutionalized, the faculty development programme needs to be more strongly embedded in the organization, and the organizational development has to be shaped, taking into account the relevant insights from organization sciences. The presented framework can be a helpful tool for this, but challenging and sharpening the professional organization development framework for sustainable high-quality medical teaching is recommended.

Sustainability of high-quality medical teaching is a future challenge. The case study has shown that, judging by the framework for organizational development, the professional role of the medical teacher has not been established at academic hospitals. High-quality medical teaching is certainly present, but in terms of sustainability, only few conditions are met organizationally, and the balance is fragile. This becomes clear at departments where financial cutbacks lead to a direct cutback on medical teaching. Also, medical research has different and richer financing sources, especially when the research is on (profitable) patient care. The professional role of the medical teacher remains at risk of falling back to a low and informal status, more than the institutionalized roles in patient care and research.

Valuable future research can be the following:

- Looking at other academic and teaching hospitals through the professional organization development framework. Other academic hospitals and teaching hospitals could benefit from the framework in terms of ‘diagnosis’ and ‘therapy’ of their organizational development.
- Evaluating the effects of the Principal Lecturer system and the attached financial bonuses on (the quality of) medical teaching. Presumably, a growth and a broader involvement in education development and research activities will be measurable. The Subsidized Innovation and Research Projects in Medical Education might have the same effect.
- Structurally evaluating how departments benefit from the faculty development and organizational development initiatives.
- Further investigating and validating the presented framework. Is it sufficient? Is it applicable in other situations (other academic and teaching hospitals)?

The presented framework appeared helpful in analyzing and understanding the impact of organizational development to support medical teaching. It offers a new perspective on faculty development, and we hope it will boost further understanding of organizational development for sustainable high-quality medical teaching.

## Essentials

- For the professional role of the medical teacher to develop, faculty development programmes need to be more strongly embedded in the organization of academic hospitals.
- The presented framework for organizational development, constructed from relevant insights from organization sciences, appears helpful in analyzing and understanding the impact of organizational development to support medical teaching.
- The presented framework can be used to ‘diagnose’ academic and teaching hospitals; it can also be used to provide ‘therapy’ in the form of suggestions for further development and improvement.
